# The Structure of a Biologically Active Influenza Virus Ribonucleoprotein Complex

**DOI:** 10.1371/journal.ppat.1000491

**Published:** 2009-06-26

**Authors:** Rocío Coloma, José M. Valpuesta, Rocío Arranz, José L. Carrascosa, Juan Ortín, Jaime Martín-Benito

**Affiliations:** 1 Centro Nacional de Biotecnología (CSIC), Campus de Cantoblanco, Madrid, Spain; 2 CIBER de Enfermedades Respiratorias (Instituto de Salud Carlos III), Recinto Hospital Joan March, Bunyola, Mallorca, Spain; Institut Pasteur, France

## Abstract

The influenza viruses contain a segmented, single-stranded RNA genome of negative polarity. Each RNA segment is encapsidated by the nucleoprotein and the polymerase complex into ribonucleoprotein particles (RNPs), which are responsible for virus transcription and replication. Despite their importance, information about the structure of these RNPs is scarce. We have determined the three-dimensional structure of a biologically active recombinant RNP by cryo-electron microscopy. The structure shows a nonameric nucleoprotein ring (at 12 Å resolution) with two monomers connected to the polymerase complex (at 18 Å resolution). Docking the atomic structures of the nucleoprotein and polymerase domains, as well as mutational analyses, has allowed us to define the interactions between the functional elements of the RNP and to propose the location of the viral RNA. Our results provide the first model for a functional negative-stranded RNA virus ribonucleoprotein complex. The structure reported here will serve as a framework to generate a quasi-atomic model of the molecular machine responsible for viral RNA synthesis and to test new models for virus RNA replication and transcription.

## Author Summary

The influenza viruses cause annual epidemics of respiratory disease and occasional pandemics that constitute a major public-health issue. The recent spillover of avian H5N1 and H1N1 swine influenza viruses to humans poses a serious threat of a new pandemic. These viruses contain a segmented RNA genome, which forms independent ribonucleoprotein particles including the polymerase complex and multiple copies of the nucleoprotein. Each of these ribonucleoprotein particles are replicated and express the encoding virus genes independently in the virus-infected cells. To better understand how these processes take place we have determined the three-dimensional structure of a model ribonucleoprotein particle that only contains 248 nucleotides of virus RNA but is biologically active in vitro and in vivo. The structure shows a circular appearance and includes 9 nucleoprotein monomers, two of which are associated to the polymerase complex. Docking of the available atomic structures of the nucleoprotein and domains of the polymerase complex has permitted us to propose a quasi-atomic model for this ribonucleoprotein particle and some of the predictions of the model have been confirmed experimentally by site-directed mutagenesis and phenotype analysis in vitro and in vivo.

## Introduction

The influenza A viruses belong to the family *Orthomyxoviridae* and are genetically and antigenically heterogeneous. They are responsible for annual epidemics of respiratory disease and represent an important public-health problem [1]. All viral subtypes can be found in their natural reservoir, that comprises several wild avian aquatic and terrestrial species. From this reservoir, influenza viruses can occasionally infect mammalian species, including man, by either gene reassortment with already established mammalian viruses or by direct adaptation [2], and thus produce a pandemic. Since 1997, transmissions of avian H5N1 influenza viruses to humans have originated hundreds of highly pathogenic infections and generated fears for a new pandemic of unprecedented impact [2],[3]. The recent transmission of swine H1N1 influenza viruses to humans could represent the first time that a new pandemic can be followed on line (http://www.who.int/csr/disease/swineflu/en/index.html). The genome of the influenza A viruses comprise eight single-stranded RNA molecules of negative polarity with partially complementary ends that form a closed structure. The native ribonucleoprotein (RNP) particles are formed by the association of these single-stranded RNAs to multiple monomers of nucleoprotein (NP) and a single copy of the polymerase, a heterotrimer composed by the PB1, PB2 and PA subunits [4],[5]. Such RNPs are independent molecular machines responsible for transcription and replication of each virus gene. When analysed structurally by electron microscopy, virion RNPs appear as flexible, supercoiled structures [6],[7]. The helical organization of the RNPs is determined by the structure of the NP, as complexes of NP and unrelated RNA also adopt helical structures [8], and purified NP can form RNP-like helical particles in the absence of RNA [9]. The polymerase complex binds the vRNA promoter, that is formed by the partially complementary 5′- and 3′-terminal sequences [10]–[12], and determines the superhelical arrangement of natural virus RNPs [13]. Although the RNPs are the essential elements for virus replication and gene expression, their structural analysis has been hampered by their heterogeneity and flexibility. However, in vivo replication of recombinant model-RNPs indicated that helical-, elliptic- or circular-shaped structures could be generated with RNA templates of diminishing lengths [14]. The clone 23 model-RNP, which represents the smallest efficient replicon, was circular in shape and showed sufficient structural rigidity to be analysed by electron microscopy and image processing after negative staining [15]. Here we report the purification of recombinant clone 23 RNPs to near homogeneity and their structural analysis by cryo-electron microscopy (cryo-EM). It is important to stress that the RNPs analysed were the final products of in vivo replication, as no RNP accumulation was observed when NP or polymerase negative mutants were used for in vivo reconstitution. The final structure shows a resolution of 12 Å for the NP and 18 Å for the polymerase complex and represents the first structure of a functional influenza virus RNP and indeed of the RNP from any negative-stranded RNA virus.

## Results

### Generation and purification of a model recombinant RNP

**Figure 1:**
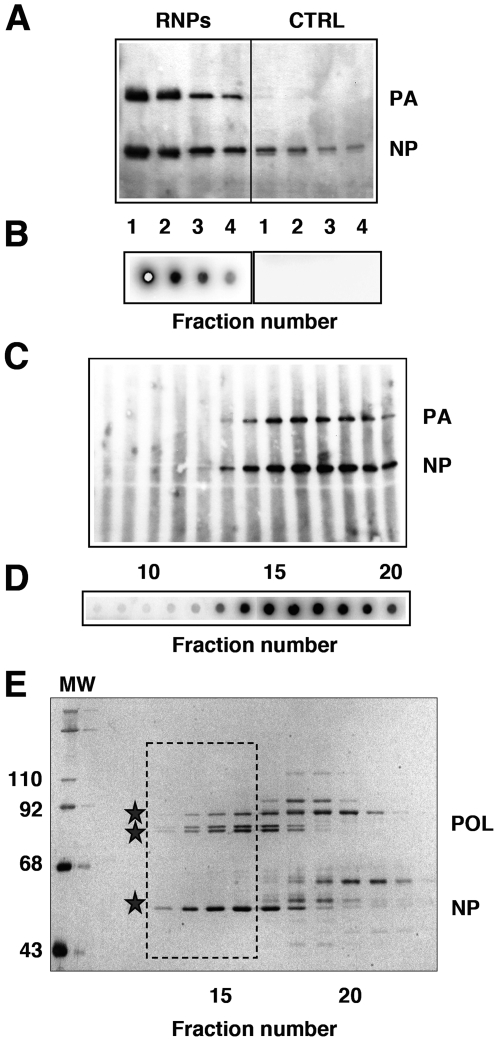
Generation and purification of a model recombinant RNP. (A) Recombinant RNPs containing a 248 nt long genomic RNA were generated and amplified in vivo as indicated under Materials and Methods. The RNPs containing a His-tagged PB2 protein (RNPs) were purified by chromatography on Ni-NTA-agarose and the eluted fractions were analysed by Western-blot using anti-PA and anti-NP antibodies. As control, untagged RNPs were generated and purified in parallel (CTRL). (B) The eluted fractions shown in (A) were assayed by in vitro transcription. (C–E) The eluted RNPs were separated on a Sephacryl S300 column and the fractions were analysed again by Western-blot (C) and in vitro transcription as above (D), as well as by silver-staining (E). The position of molecular weight markers (MW) is indicated to the left. The stars indicate the position of the polymerase subunits (POL) and the nucleoprotein (NP). The frame indicates the fractions chosen for electron microscopy analyses.

Previously, we used recombinant RNPs purified by successive glycerol gradient centrifugation steps to analyse their structure by electron microscopy of negative-stained samples [15]. To improve the purity and yield of the RNP preparations, we used a PB2 subunit containing a His-tag at the C-terminus, a modification that did not alter the in vivo replication activity of the RNPs, as described previously [16]. The purification protocol involved an optimised Ni-NTA-agarose affinity step, a gel-filtration chromatography and a final concentration on a Ni-NTA-agarose resin. Such procedure allowed the routine preparation of essentially homogeneous and biologically active RNPs with a concentration appropriate for structural analysis (Fig. 1A–E). Most of the cellular contaminants could be removed in the first Ni-NTA column, while active RNPs were concentrated (Fig. 1A, B). The remaining contaminants were eliminated by gel filtration (Fig. 1E), a step in which the signals for the polymerase and NP co-migrated with the in vitro transcriptional activity (Fig. 1C, D). The purified RNPs (Fig. 1E, frame) were concentrated by binding to and elution from Ni-NTA-agarose (data not shown) and used for cryo-EM.

### Cryo-electron microscopy structure of a model recombinant RNP

To generate an initial model for reconstruction, a purified RNP sample was stained with uranyl-acetate and imaged at 20° tilt in a FEI Tecnai G^2^ field emission gun microscope. A total of 2035 particle images were employed to generate a three-dimensional reconstruction using the SPIDER algorithms [17]. To generate a three-dimensional reconstruction of frozen-hydrated RNPs, samples of purified RNPs (Fig. 1E, frame) were fast-frozen on holey-grids and imaged in the same microscope. A total of 9571 individual particle images were selected from the micrographs after CTF correction and used for refinement (see Fig. S1 for a gallery of single particle images). Two independent refinement processes were carried out, with and without imposing 9-fold symmetry. The three-dimensional reconstruction obtained by imposing 9-fold symmetry lacked information about the polymerase complex but could achieve better resolution for the NP ring. On the contrary, refinement without imposing symmetry allowed reconstruction of the complete RNP particle but the resolution obtained was significantly lower (Fig. S2).

**Figure 2:**
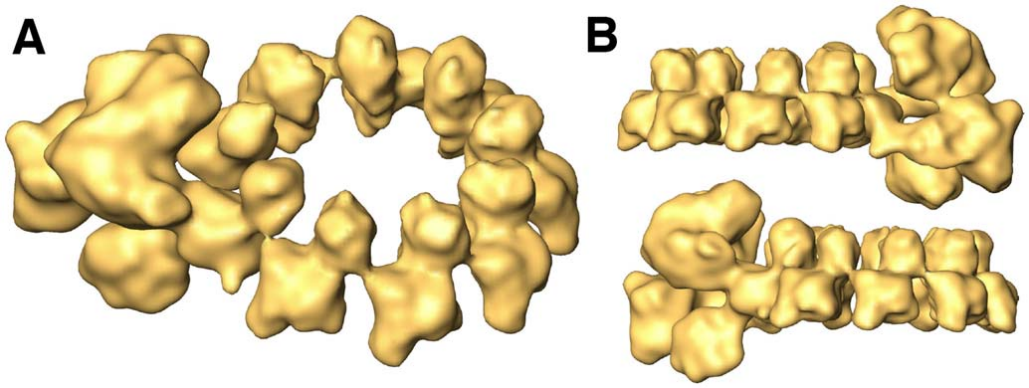
Three-dimensional model of a recombinant virus mini-RNP. The final volume is a chimera containing the polymerase and two adjacent NP monomers derived from a non-symmetrical volume and the rest of the NP ring derived from the symmetrical volume (see Fig. S2). (A) Perspective view of the composite three-dimensional structure for the recombinant RNP. (B) Side views.

The final structure is shown in Fig. 2 and Video S1, and represents a composite map formed by the 7 NP monomers not contacting the polymerase, that are derived from the structure refined with symmetry, while the polymerase complex, as well as the 2 adjacent NP monomers are derived from the volume refined without symmetry. Therefore, the resolutions for either section of the model are different: 12 Å for the NP ring and 18 Å for the polymerase complex (Fig. S3). Each NP monomer consists of two domains, an upper head domain and a centred body, which contains a small mass protruding at the bottom. When represented at the calculated threshold no massive contacts among the NP monomers were apparent, suggesting that the interaction sites are flexible or random coil chains. The polymerase complex is in contact with two of the NP monomers, which lack apparent interaction with each other (Fig. 2).

### Docking defined subunit domains in the structure of the polymerase complex

**Figure 3:**
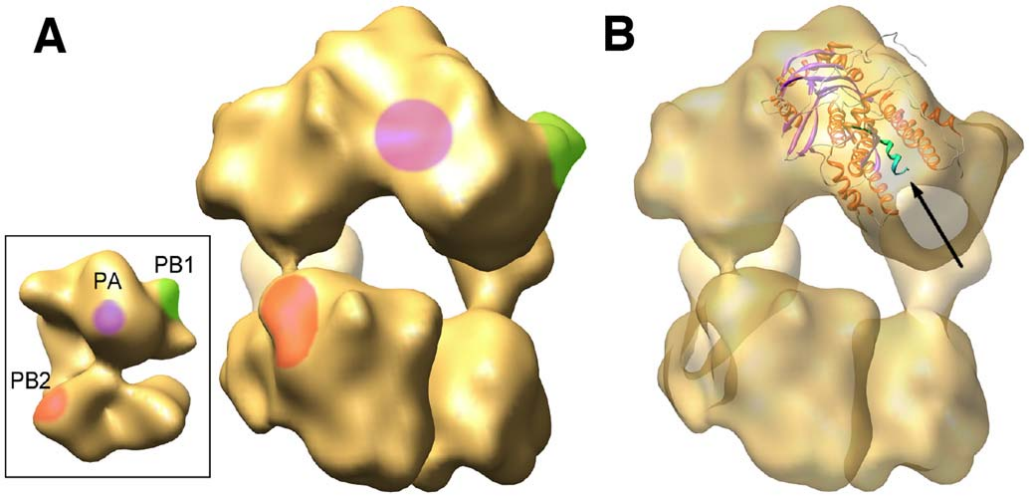
Docking the atomic structure of PA-PB1 complex into the RNP structure. (A) The insert shows the three-dimensional model for the virus polymerase complex present in the RNP as reported by Area et al. [16]. The handedness of the structure has been reversed as compared to the one published, as indicated by the docking of the atomic structure of the NP (see Supporting online material). The location of specific domains in the PB1 (green), PB2 (red) and PA (violet) subunits are indicated. A front-view of the polymerase present in the RNP cryo-EM structure is presented, with the locations of the polymerase domains as inferred from the negative-stained model. (B) The same front-view of the polymerase is presented with the docking of the PA(C)-PB1(N) dimer. The N-terminal PB1 peptide is indicated with an arrow and highlighted in green.

The structure of the polymerase complex resembles that previously obtained by negative-staining [16],[18], but has higher resolution. A comparison between both structures allowed the localisation of specific subunit domains, as defined earlier by binding of monoclonal antibodies or tagging (Fig. 3A) and suggest that the main NP-polymerase interactions are mediated by the PB1 and PB2 subunits. These interactions are quite different in intensity, the former being stronger than the latter (Fig. 2, Fig. S2 and Video S1). Docking the recently reported atomic structure of the PA(C)-PB1(N) dimer [19],[20] was consistent with its predicted localisation (Fig. 3B) [16] and would suggests that the PB1 and PA subunits account for the upper, bulkier section of the complex while PB2 would be localised at the bottom region.

### Docking the NP atomic structure in the NP ring

We also carried out a docking of the atomic structure of the NP in the cryo-EM reconstruction. The handedness of the cryo-EM map was determined on the basis of the correlation coefficient of the NP atomic structure docked into the symmetrised volume. The fitting assays were carried out with both handednesses, using either volumetric or laplacian criteria. The maximum correlation coefficients were 0.854 and 0.341 for volumetric and laplacian tests, respectively. These values were 2 to 30% better for the selected as compared to the alternative handedness. In addition, another important consideration indicates that the selected handedness is correct. In the atomic structure of the influenza NP (pdb accession number 2IQH) there are some portions of the molecule that are not defined. The connections between the loop 402–428 (which is involved in NP-NP interaction; see below) and the body of the molecule could not be determined (sequence A428-S438). The distance between these two amino acids in the selected fitting was around 25 Å, compatible with a 10 amino acids distance, whereas in the fitting performed in the structure with the opposite handedness, these two amino acids were 41 Å apart.

**Figure 4:**
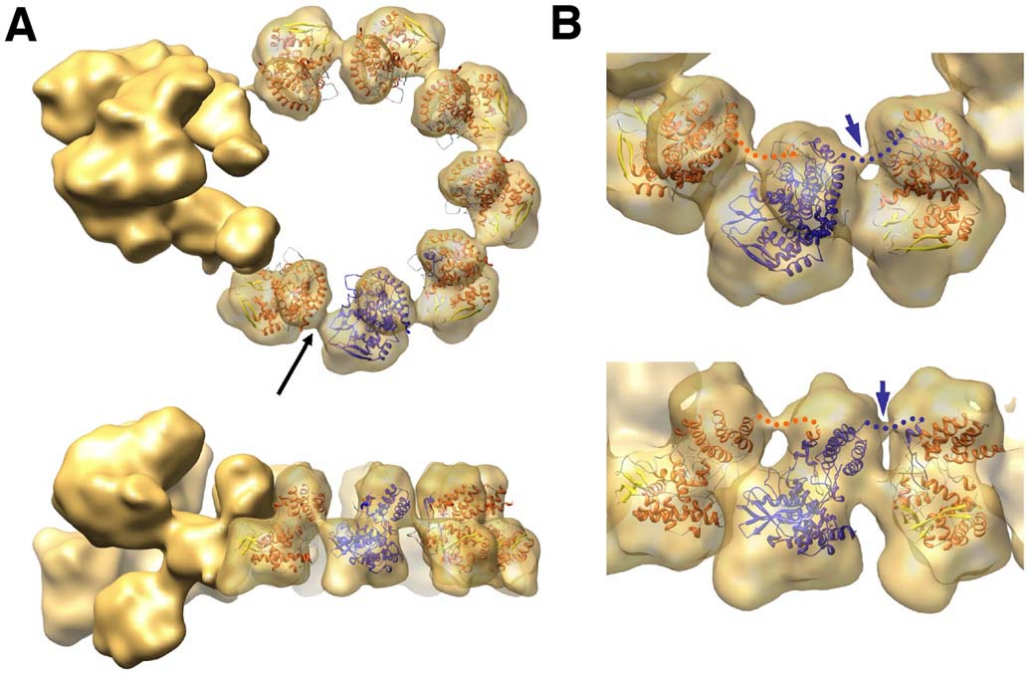
Docking the atomic structure of NP monomers into the RNP model. (A) The atomic structure of NP [21] is represented within the volume of the NP monomers derived from a reconstruction obtained imposing 9-fold symmetry. Upper and side views are presented. One of the monomeric atomic structures is highlighted in blue to reveal the localisation of the connecting loop within the neighbouring monomer. The black arrow points to the potential RNA connection among NP monomers. (B) Close-up view of three NP monomers represented at σ = 1.5. Upper and perspective views are shown. The blue arrow points to the connection between the NP monomers at the top of the molecules. The presumptive connection of the NP head and the loop inserted in the neighbouring monomer is indicated by a dotted line.

The result of the docking is shown in Fig. 4A and confirms the quality of the structural model obtained. A good fit is observed between the two domains described in the atomic structure and the volume of the NP monomer. However, additional masses are observed at the top and at the bottom of each NP monomer. It could be argued that such additional masses arise as a consequence of using an initial negative-stain model that was derived by conical-tilting. However, we used the same image data set to carry out a control refinement, using as initial model a 9-mer-ring structure generated with the atomic model of the NP filtered to a resolution of 30 Å, and the final model obtained was indistinguishable from that shown in Fig. 2 (data not shown). Furthermore, the angular coverage of the images (Fig. S4) was sufficient to exclude the missing cone as an explanation for this extra volume. Thus, we feel that the additional masses detected in the cryo-EM model of the NP monomers are bona fide.

**Figure 5:**
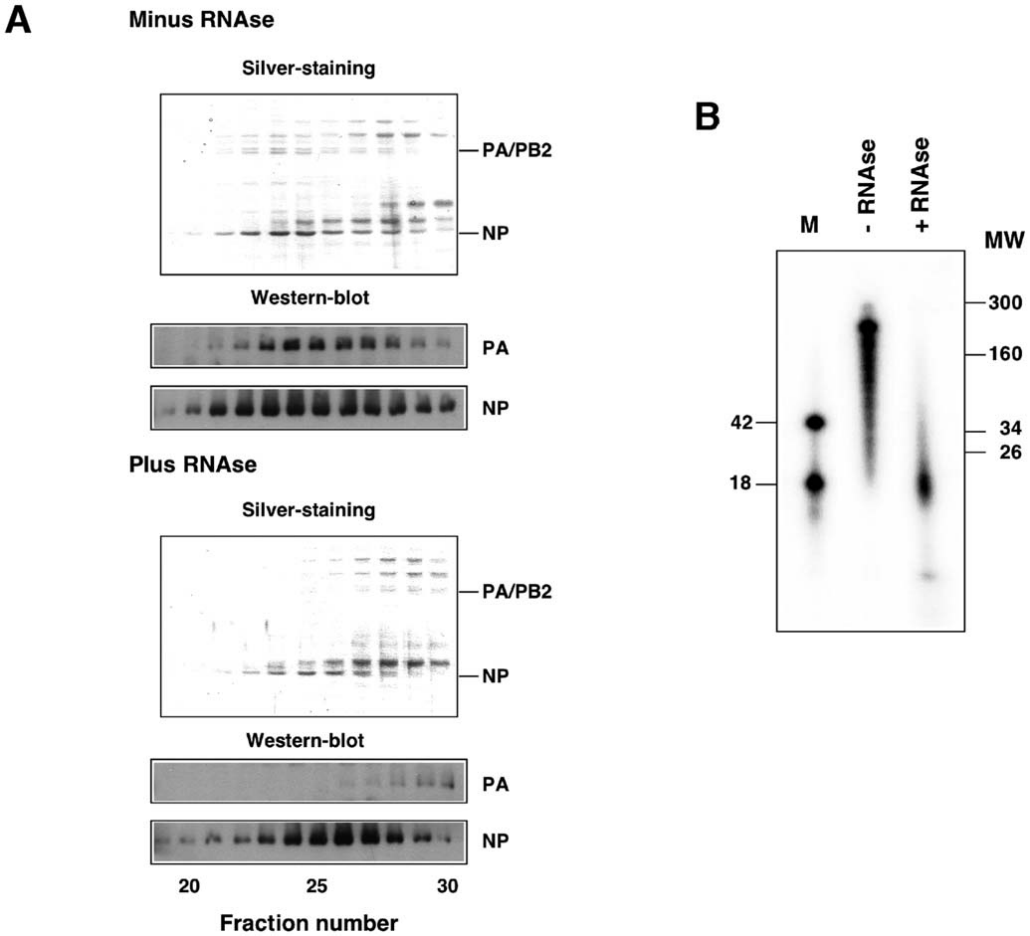
Probing the RNP structure by RNAse treatment. Recombinant RNPs were generated and amplified in vivo as described in Fig. 1. After purification by affinity chromatography on Ni-NTA-agarose, the RNPs were treated with a mixture of pancreatic RNAse (1.2 mg/ml) and T1 RNAse (30 u/ml) for 30 min at room temperature. As a control, the purified RNPs were similarly incubated in the absence of any RNAse. (A) The RNPs were filtered on a Sephacryl S300 column as indicated in Fig. 1 and each fraction was analysed by Western-blot using antibodies specific for NP or PA. The position of NP and PA is indicated to the right. (B) The RNA present in the RNAse-treated or mock-treated RNPs was extracted, terminally labelled with γ-32P-ATP and analysed on a 12% polyacrylamide-urea denaturing gel. Labelled oligonucleotides of 42 and 18 nt in length were run in parallel (M). The mobility of molecular weight markers is indicated to the right.

We propose that the extra mass at the top of the NP monomer corresponds to the protein sequences not solved in the crystal structure [21] while that at the bottom may contain genomic RNA. In fact, when decreasing threshold values were used to represent the RNP volume, the additional mass at the bottom of the NP was persistent, suggesting a high mass density (data not shown). To test the potential RNA-dependence of the RNP structure, these were purified by affinity chromatography on Ni-NTA-agarose, extensively treated with T1 and pancreatic RNAses and analysed by gel filtration. The results are shown in Fig. 5A and clearly indicate that the interaction between the polymerase complex and the NP ring is highly RNA-dependent, as both substructures could be separated after RNAse treatment. On the other hand, the size of the template RNA before and after digestion with RNAse was analysed and a resistant band of around 18 nt was apparent (Fig. 5B). Since an average content of 24 nt per NP monomer has been determined [14], this result would suggest that most of the template RNA is uniformly distributed along the RNP structure and protected by association to the NP.

Docking of the atomic structure of the NP monomers into the cryo-EM structure also allowed us to predict their interaction interfaces. It was earlier proposed that interaction of the loop 420 (positions 402–428) with a neighbouring NP monomer would account for NP polymerisation [21], but this was suggested on the basis of the formation of a crystallographic trimer and no functional data was reported. The interaction among NP monomers is conserved in the NP docking presented here, with the only need to alter the angle between NP monomers from about 120° in the crystal to 40° in the RNP volume (Fig. 4A). This interaction interface would be more realistic, as no NP trimeric structure has been described in natural virus RNPs, and would imply a small change in the arrangement of the connections between the loop and the body of the NP (positions 428–438 and 396–402). These connections are in any case highly flexible and were not resolved in the atomic structure of the trimer [21]. Although such a flexible connection is not detectable in the cryo-EM map at the threshold shown in Fig. 4A (σ = 2.5), it is clearly visible when the volume is represented at σ = 1.5 (Fig. 4B, blue arrow).

### Functional relevance of the predicted NP-NP contact sites

**Figure 6:**
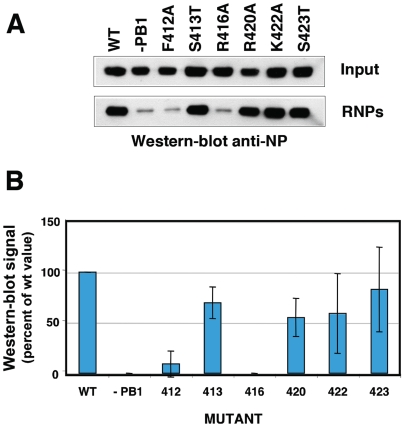
Phenotype of RNPs with NP mutations in the NP-NP interaction site. Recombinant RNPs were generated and amplified in vivo using either wt of mutant NP as indicated. After purification of progeny RNPs by pull-down with Ni-NTA-agarose, their accumulation was determined by Western-blot with anti-NP antibodies. (A) Results of a representative experiment, including the analysis of total cell extracts (Input) and the purified RNPs (RNPs). (B) Average and range of two experiments.

**Figure 7:**
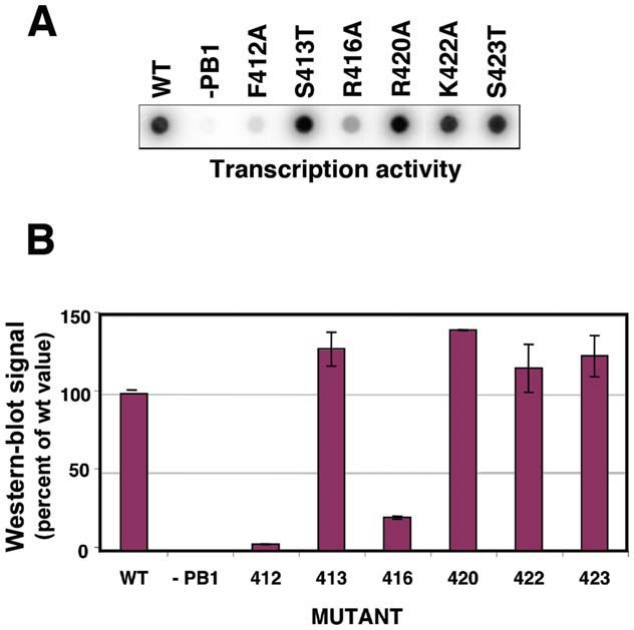
Replication in vivo of RNPs containing wt or mutant NP. The assay for in vivo replication was performed as described in Materials and Methods and the legend to Figure 6. The concentration of RNPs after purification by affinity chromatography was determined by in vitro transcription. (A) Results of a representative experiment, including the analysis of total cell extracts (Input) and the purified RNPs (RNPs). (B) Average and range of two experiments.

It is not clear whether the contacts between the NP monomers observed in the atomic structure of the trimer would be strictly conserved in the functional RNP nonameric structure. Hence we mutated several of the positions in the loop, affecting either conserved or non-conserved amino acids (Fig. S5), and tested the biological activity of the RNP. The replication of a viral RNP does not lead to a naked progeny RNA but rather a progeny RNP structure and it is generally accepted that encapsidation of the newly synthesised RNA by the polymerase complex and NP monomers is coupled to RNA replication. Hence, if the mutations were to affect the NP-NP interaction, a deficiency in RNP replication would be expected. Thus we reconstituted in vivo mini-RNPs by transfection of plasmids encoding the polymerase subunits (of which PB2 as a His-tagged protein), a clone 23 template RNA and either wt or mutant NP, and purified them by Ni-NTA-agarose chromatography. The accumulation of progeny RNPs was determined by Western-blot using anti-NP antibodies and represents the in vivo replication phenotype. Mutants R416A and F412A were strongly affected in replication, whereas mutants S413T, F420A, K422A and S423A behaved as wt (Fig. 6A, B and Fig. S5). These results confirm the relevance of the interaction between R416 in the loop and E339 in the connecting NP [21] and suggest that residue F420 in the loop does not play an important role in the interaction. On the other hand, the phenotype of mutant F412A indicates that it is important for viral RNA replication. To further analyse the phenotype of the NP mutants generated, the amount of purified mutant RNPs recovered by replication in vivo was determined by measuring their in vitro transcription activity. The results of a typical experiment are presented in Fig. 7A and average of two independent experiments is shown in Fig. 7B. These results are consistent with the deficiency in the replication activity observed for mutants R416A and F412A.

**Figure 8:**
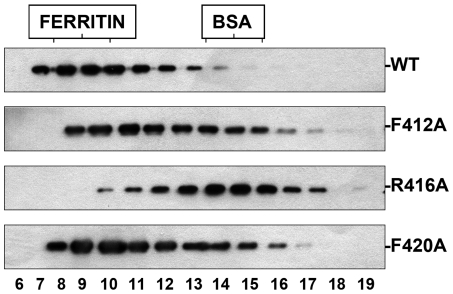
Homopolymerisation of wt or mutant NP. The aggregation state of wt or mutant NPs was determined by gel filtration. Cultures of COS1 cells were transfected with plasmids expressing either wt of mutant NP, as indicated, and total cell extracts were treated with RNAse and filtered on a Sephacryl S300 column. The eluted fractions were analysed by Western-blot with anti-NP antibodies. The position of ferritin (440 kDa) and bovine serum albumin (BSA; 67 kDa) is shown on the top of the Figure.

The replication-defective phenotype observed for these mutants could be the consequence of a defect in their homopolymerisation capacity. To analyse this possibility mutant or wt NP were expressed by transfection in COS1 cells and total extracts were analysed by gel-filtration after extensive RNAse treatment. Under these conditions, wt NP formed large complexes compatible with NP polymers. On the contrary, mutant R416A, that was shown as negative in NP-NP association [21], behaved as monomer (Fig. 8). The phenotype of the other mutants correlated with their replicative activity in vivo. Thus, mutant F420A behaved as wt while mutant F412A showed an intermediate phenotype.

## Discussion

In this report we have presented the three-dimensional structure of an active influenza virus RNP, as determined by cryo-EM. In fact, this represents the first structure of a biologically active RNP from any negative-stranded RNA virus. Two technical developments have allowed this breakthrough: (i) the generation of recombinant RNPs that are efficient replicons and have sufficient structural rigidity [14] and (ii) the optimisation in the purification protocols of RNPs amplified in vivo. As compared to full-length virion RNPs, the structure reported here would represent a minimal RNP in which the helical section has been deleted and only the promoter region bound to the polymerase complex and the terminal loop remains. The structure obtained for the polymerase complex present in the RNP is compatible with those reported earlier by negative-staining [16],[18] and represents the most accurate model for a complex polymerase of a negative-stranded RNA virus thus far reported. The correlation with the sites previously mapped [16] and the docking of the atomic structure of specific domains permitted the rough localisation of the polymerase subunits (Fig. 3). Unfortunately, the other polymerase domains whose atomic structure is known [22]–[24] are not large and conspicuous enough to allow unambiguous docking in the cryo-EM structure.

The interaction among NP monomers was analysed by docking of the atomic structure into the NP ring. The model obtained is compatible with the interaction mode proposed earlier [21] and further indicated that additional side-by-side interactions are now possible due to the tighter packing of the monomers (see Fig. 4A, black arrow). The relevance of the 420–428 loop in the NP-NP interaction was verified functionally: The contacts of amino acid R416 and F412 are essential for replication, while amino acid K422 does not appear to be important, in spite of being conserved among type A and B viruses (Fig. S5). Previous biochemical studies had shown that residue R416 is involved in NP-NP interaction [25] and that both F412 and R416 were important for RNA binding [26]. In view of the results presented here it is possible that the RNA-binding defect detected might be secondary to the homopolymerisation failure. In addition, the residue at position 412 appears to be important for the template activity of the RNP, since mutation F412A specially affected the in vitro transcription of mutant RNPs (compare Figs. 5 and 6).

Contrary to the N-RNA complexes in the Mononegavirales [27],[28], that contain 9 nucleotides associated to each N molecule, we have estimated an average of 24 nucleotides per NP monomer in influenza RNPs [14]. The structure of the RNP presented here is compatible with the RNA-binding site being located at the groove between the head and body domains in the NP, as previously suggested [21],[29]. Indeed, a connecting mass is apparent in the appropriate position (see Fig. 3A, black arrow) that could represent the template RNA in addition to protein contacts. However, most of the RNA sequence present in the RNP is resistant to extensive RNAse treatment and the main protected fragment is around 18 nt long (Fig. 5). This would suggest that the template RNA is distributed uniformly along the RNP structure, i.e. variations of the average value of 24 nt per NP monomer are small. Furthermore, the size of the protected fragment (18 nt) is similar to the average assignment of RNA per NP, suggesting that the template RNA associates to several regions of the NP and could contribute to the extra mass observed at the bottom of each NP monomer. In addition, the N-terminal region of NP, which has been implicated in binding to RNA by biochemical assays [30] and is not represented in the atomic structure of the protein [21],[29], could also contribute to this extra mass.

In summary, we have reported the first structure of a biologically active influenza RNP. This three-dimensional structure reveals the NP-NP interaction domain and will serve as a framework to generate a quasi-atomic model of the molecular machine responsible for viral RNA synthesis.

## Materials and Methods

### Biological materials

The origin of plasmids pGPB1, pGPB2His, pGPA, pGNP(polyA) and pT7ΔNSRT clone 23, containing sequences derived from the A/Victoria/3/75 influenza virus strain, has been described [14],[16],[31]. The vaccinia recombinant virus expressing T7 RNA polymerase (vTF7-3) [32] was provided by B. Moss. The origin of antibodies specific for PB1, PB2 and PA has been described [14],[33],[34]. Antibodies specific for NP were generated by immunisation of rabbits with purified His-NP. The NP mutants were generated by site-directed mutagenesis on pGNP(polyA) plasmid using the Stratagene Quickchange kit and specific oligonucleotides (sequences available upon request) and their genotype was verified by sequencing.

### Amplification and purification of recombinant RNPs

Recombinant RNPs containing the ΔNS clone 23 genomic RNA (248 nt) were generated and amplified in vivo by transfection of plasmids pGPB1, pGPB2His, pGPA, pGNP(polyA) and pT7ΔNSRT clone 23 into vaccinia vTF7-3-infected COS1 cells as described previously [16]. For RNP purification, the clarified cell extracts were incubated overnight at 4°C with Ni-NTA-agarose resin in a buffer containing 50 mM Tris-HCl-100 mM KCl-5 mM MgCl2-0.5% Igepal-20 mM imidazol-1 u/µl RNAsin-EDTA-free protease inhibitors cocktail, pH 8. The resin was washed with 80 volumes of 50 mM Tris-HCl-100 mM KCl-5 mM MgCl2-0.5% Igepal-20 mM imidazol, pH 8 and 20 volumes of the same buffer containing 50 mM imidazol. Finally, the RNPs were eluted with 50 mM Tris-HCl-100 mM KCl-5 mM MgCl_2_-0.5% Igepal-150 mM imidazol, pH 8. The eluted RNPs were filtered on a Sephacryl S300 column equilibrated with 50 mM Tris-HCl-100 mM KCl-5 mM MgCl2-0.5% Igepal-20 mM imidazol, pH 8 and the peak RNP fractions were further bound to Ni-NTA-agarose in 50 mM Tris-HCl-100 mM KCl-5 mM MgCl2-0.5% Igepal-20 mM imidazol-1 u/µl RNAsin-EDTA-free protease inhibitors cocktail, pH 8, washed once with 50 mM Tris-HCl-100 mM KCl-5 mM MgCl2-0.5% Igepal-20 mM imidazol, pH 8 and eluted with 50 mM Tris-HCl-100 mM KCl-5 mM MgCl_2_-0.3% CHAPS-150 mM imidazol, pH 8.

### Biochemical techniques

Western-blotting was performed as described [14]. Protein silver-staining was carried out as indicated before [35]. To determine the transcription activity of purified RNPs, samples were incubated in a buffer containing 50 mM Tris-HCl-2 mM MgCl2-100 mM KCl-1 mM DTT-10 µg/ml actinomycin D-1 u/µl RNAsin-1 mM ATP-1 mM CTP-1 mM UTP-10 µM α-P^32^-GTP (20 µCi/µmol)-100 µM ApG for 60 min at 30°C. The RNA synthesised was TCA precipitated, filtered through a nylon filter in a dot-blot apparatus and quantified in a phosphorimager.

To test the in vivo RNP replication, cultures of COS1 cells were infected with vaccinia vTF7-3 and transfected with plasmids pGPB1, pGPB2His, pGPA, pGNP(polyA) (or mutants thereof) and pT7ΔNSRT clone 23. Total cell extracts were used for purification by affinity chromatography on Ni-NTA-agarose as indicated above and the accumulation of progeny RNPs was determined by Western-blot with anti-NP-specific antibodies and by measuring their in vitro transcription activity.

To determine the NP aggregation state, cultures of COS1 cells were infected with vaccinia vTF7-3 and transfected with plasmid pGNP(polyA) (or mutants thereof). Total cell extracts were prepared, treated with 50 µg/ml of RNAse A for 2 hours at room temperature and analysed by filtration over a Sephacryl S300 column calibrated with ferritin (440 kDa) and BSA (67 kDa).

### Electron microscopy and image processing

For electron microscopy of negatively stained samples 4 µl aliquots of purified RNPs were applied to glow-discharged carbon grids for 1 min and then stained for 1 min with 2% uranyl acetate. Low-dose images were taken on a 200 kV FEI Tecnai G^2^ Field emission gun electron cryomicroscope operated at a nominal magnification of 50 k at 20° tilt. A total of 2035 individual RNP images were extracted and processed to generate an initial model using the SPIDER software [17].

For cryoelectron microscopy, 5 µl aliquots of purified RNPs were applied to glow-discharged Quantifoil holey grids for 2 min, blotted and frozen rapidly in liquid ethane at −180°C. Images were taken with the same conditions as in the negative stain experiments but without tilting. The selected micrographs were scanned on a Zeiss scanner (Photoscan TD, Z/I Imaging Corporation) with a final pixel size corresponding to 2.8 Å. Contrast transfer function (CTF) of micrographs was estimated using ctffind software [36] and corrected using Bsoft [37]. A total of 9571 images were subjected to two independent refinements with and without imposing 9-fold symmetry using SPIDER software [17]. After reaching the convergence of these refinements, the reconstructions yielded resolutions of 18 and 12 Å for non-symmetrized and symmetrized structures, respectively (FSC 0.3 criterion). The final tilt range assigned in the refinement for the whole set of individual images was checked (Fig. S5) and showed an angular distribution where the effect of missing cone in the reconstruction could be considered as negligible.

The absolute handedness of the volumes was determined using the atomic structure of NP protein [21], and turned out to be the opposite to that previously published [15],[16]. Docking experiments were carried out using SITUS software [38]. Finally, and to verify the positions of the extra mass and the quality of the three-dimensional reconstruction, an additional refinement was carried out using as initial model the structure of the 9 NP-mer ring resulting from the docking experiments, filtered at 35 Å. This refinement yielded a reconstruction similar to the final structure presented here, showing that the additional masses detected in the cryo-EM structure protruding from the NP monomers are *bona fide*. Volume handling was carried out using XMIPP software [39] and general visualization was performed using Chimera [40] and Amira (http://amira.zib.de). The cryo-EM map has been deposited in the Electron Microscopy Data Bank (accession code EMD-1603) and the fitted atomic structure in the Protein Data Dank (accession code 2wfs).

## Supporting Information

Figure S1 Gallery of images. (A) Examples of images derived from negative-stained samples used to generate the initial model for reconstruction. (B) Images derived from frozen samples. The corresponding projections of the final volume are presented to the left to help in the identification.
(2.60 MB TIF)

Figure S2 Three-dimensional models generated by refinement with and without imposed 9-fold symmetry. (A) Volumes obtained after refinement without imposed symmetry. (B) Volumes obtained after refinement with imposed 9-fold symmetry. Images at the top show upper views while images at the bottom are lower views of the structures.
(3.90 MB TIF)

Figure S3 Determination of resolution. The Fourier shell correlation is presented as a function of the normalised frequency for the reconstruction imposing 9-fold symmetry (blue) or without imposing any symmetry (red). The inverse of the corresponding resolution is indicated below each frequency value. The green line denotes the FSC = 0.3 cut-off.
(0.51 MB TIF)

Figure S4 Assignment of tilt in three-dimensional reconstruction. The distribution of final tilt assigned to the complete set of images after refinement is presented.
(0.63 MB TIF)

Figure S5 Mutations to analyse the NP-NP interaction site. (A) The atomic structure of influenza NP is shown in an orientation appropriate to see the protruding loop present around position 420 in the sequence. The relevant amino acids in the loop are highlighted. (B) The mutations introduced in the loop are indicated, including those involving non-conservative changes in conserved positions (in red) and conservative changes in non-conserved positions (in black). (C) Alignment of the relevant sequence for influenza viruses of the A, B and C types indicating the same mutations indicated in panel B.
(1.60 MB TIF)

Video S1 Movie of the RNP structure.
(8.08 MB MOV)
