# Supplementary figures and images for: The Structure of a Biologically Active Influenza Virus Ribonucleoprotein Complex
Source: PLoS Pathog. 2009 Jun 26;5(6):e1000491. doi: 10.1371/journal.ppat.1000491 (PMC2695768; doi:10.1371/journal.ppat.1000491)

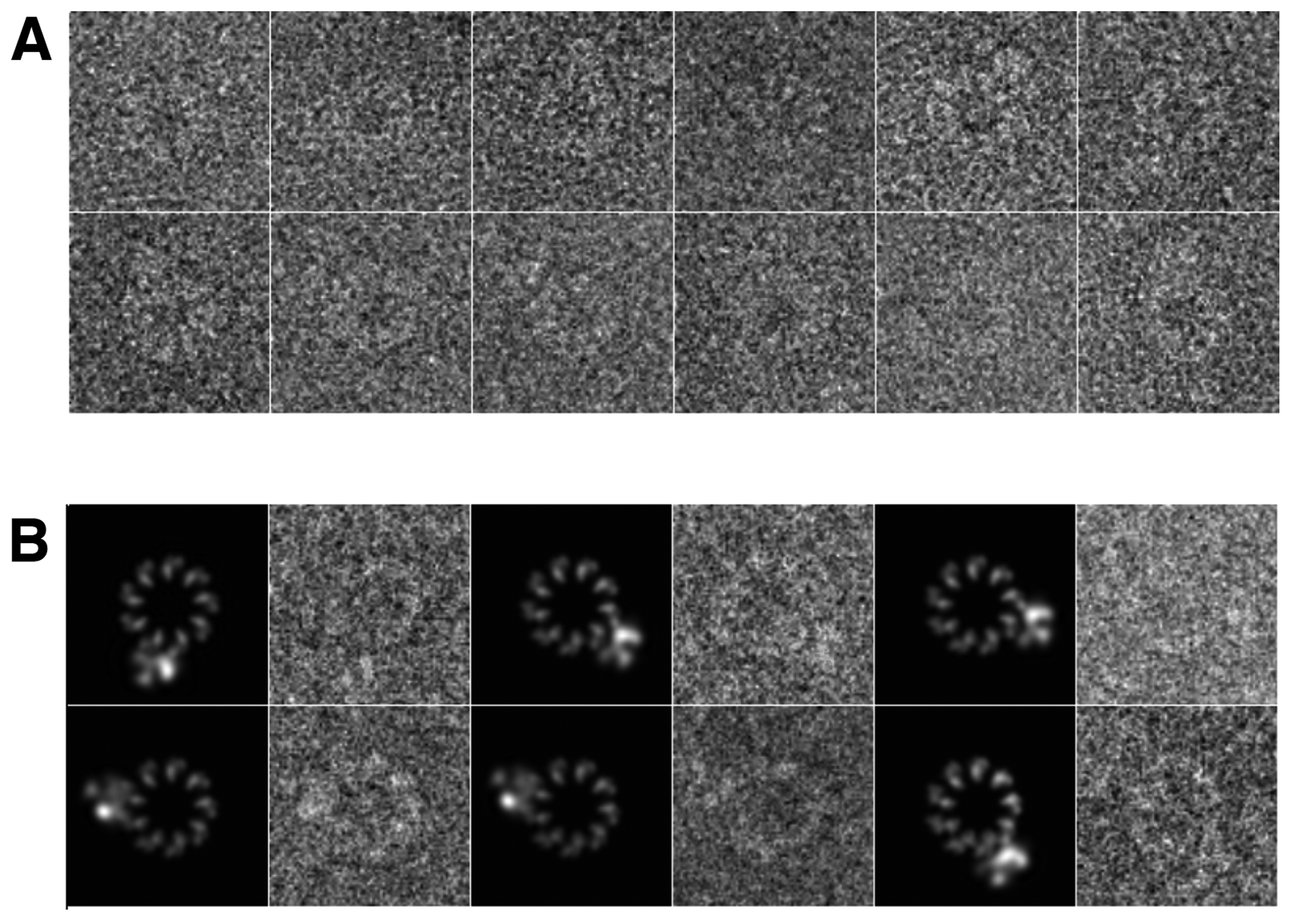

Supplement: Figure S1 — Gallery of images. (A) Examples of images derived from negative-stained samples used to generate the initial model for reconstruction. (B) Images derived from frozen samples. The corresponding projections of the final volume are presented to the left to help in the identification. (2.60 MB TIF) [file ppat.1000491.s001.tif]

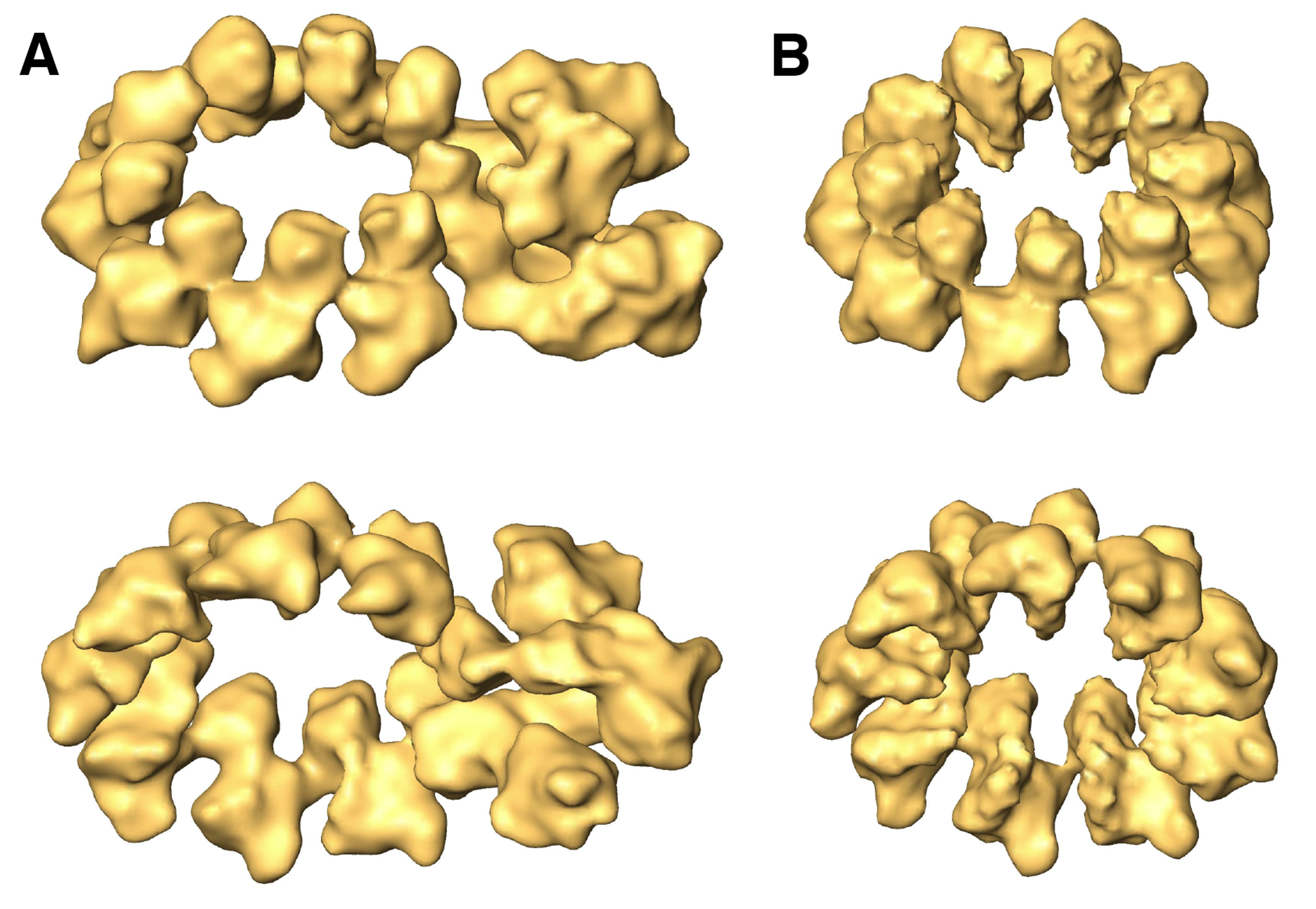

Supplement: Figure S2 — Three-dimensional models generated by refinement with and without imposed 9-fold symmetry. (A) Volumes obtained after refinement without imposed symmetry. (B) Volumes obtained after refinement with imposed 9-fold symmetry. Images at the top show upper views while images at the bottom are lower views of the structures. (3.90 MB TIF) [file ppat.1000491.s002.tif]

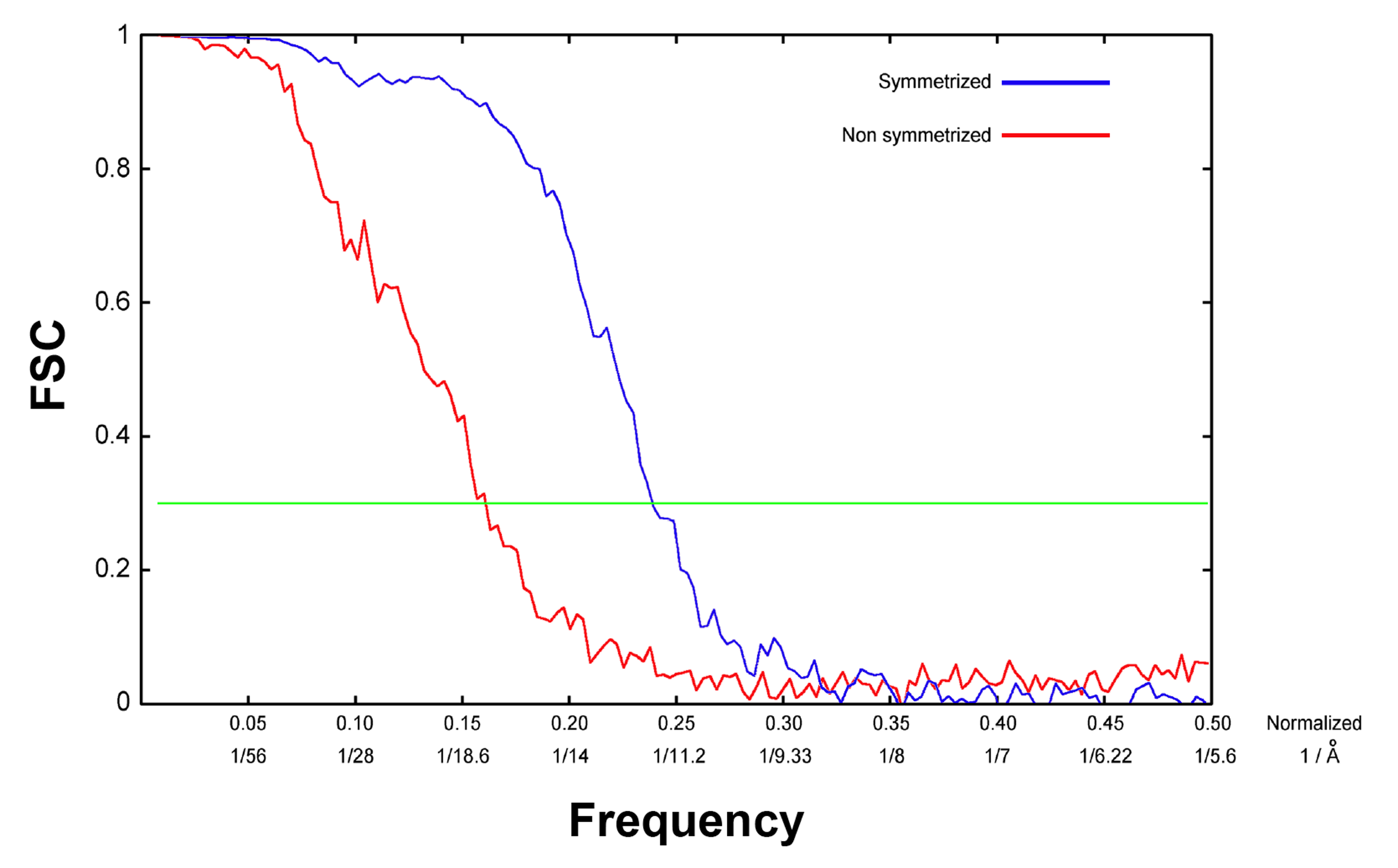

Supplement: Figure S3 — Determination of resolution. The Fourier shell correlation is presented as a function of the normalised frequency for the reconstruction imposing 9-fold symmetry (blue) or without imposing any symmetry (red). The inverse of the corresponding resolution is indicated below each frequency value. The green line denotes the FSC = 0.3 cut-off. (0.51 MB TIF) [file ppat.1000491.s003.tif]

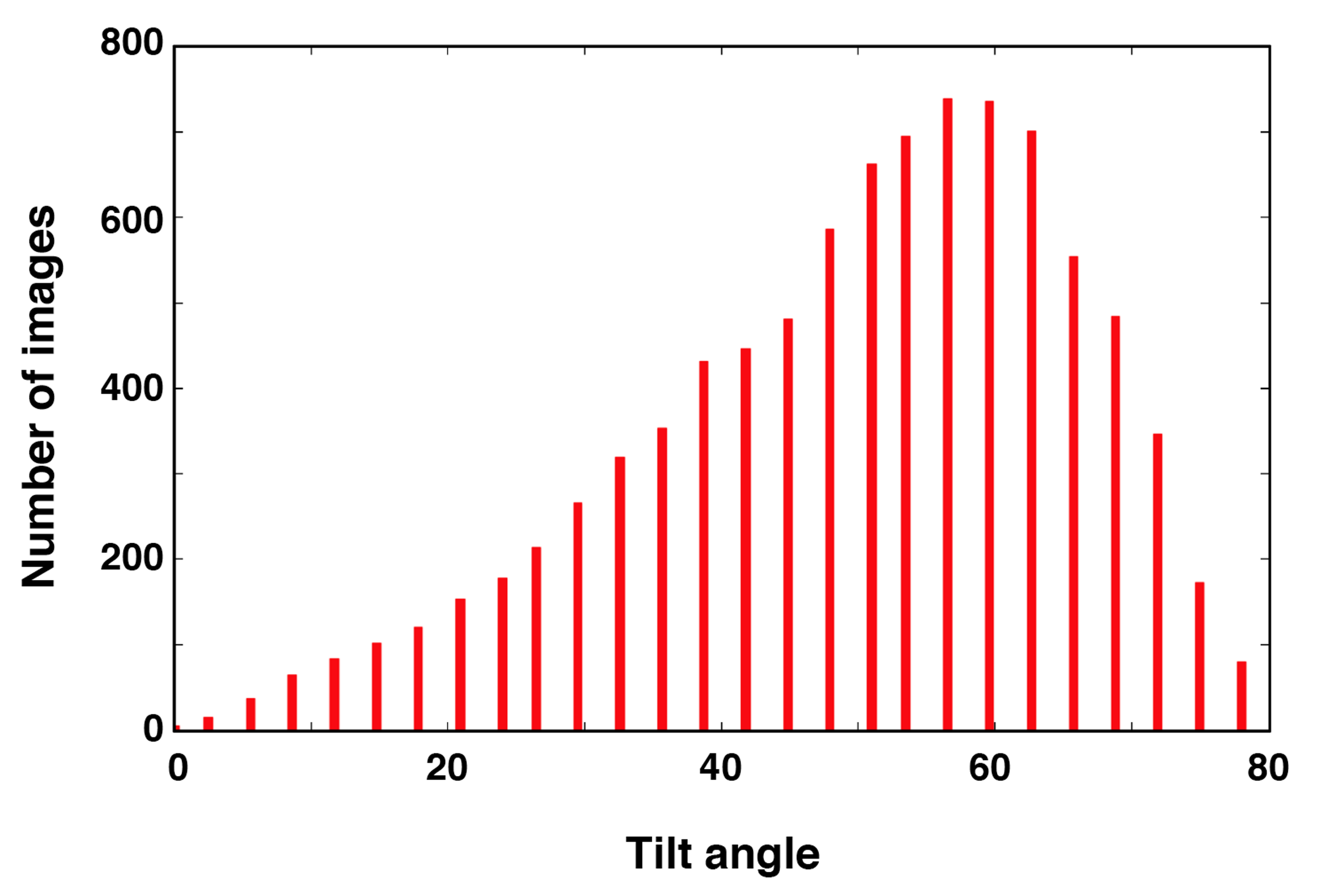

Supplement: Figure S4 — Assignment of tilt in three-dimensional reconstruction. The distribution of final tilt assigned to the complete set of images after refinement is presented. (0.63 MB TIF) [file ppat.1000491.s004.tif]

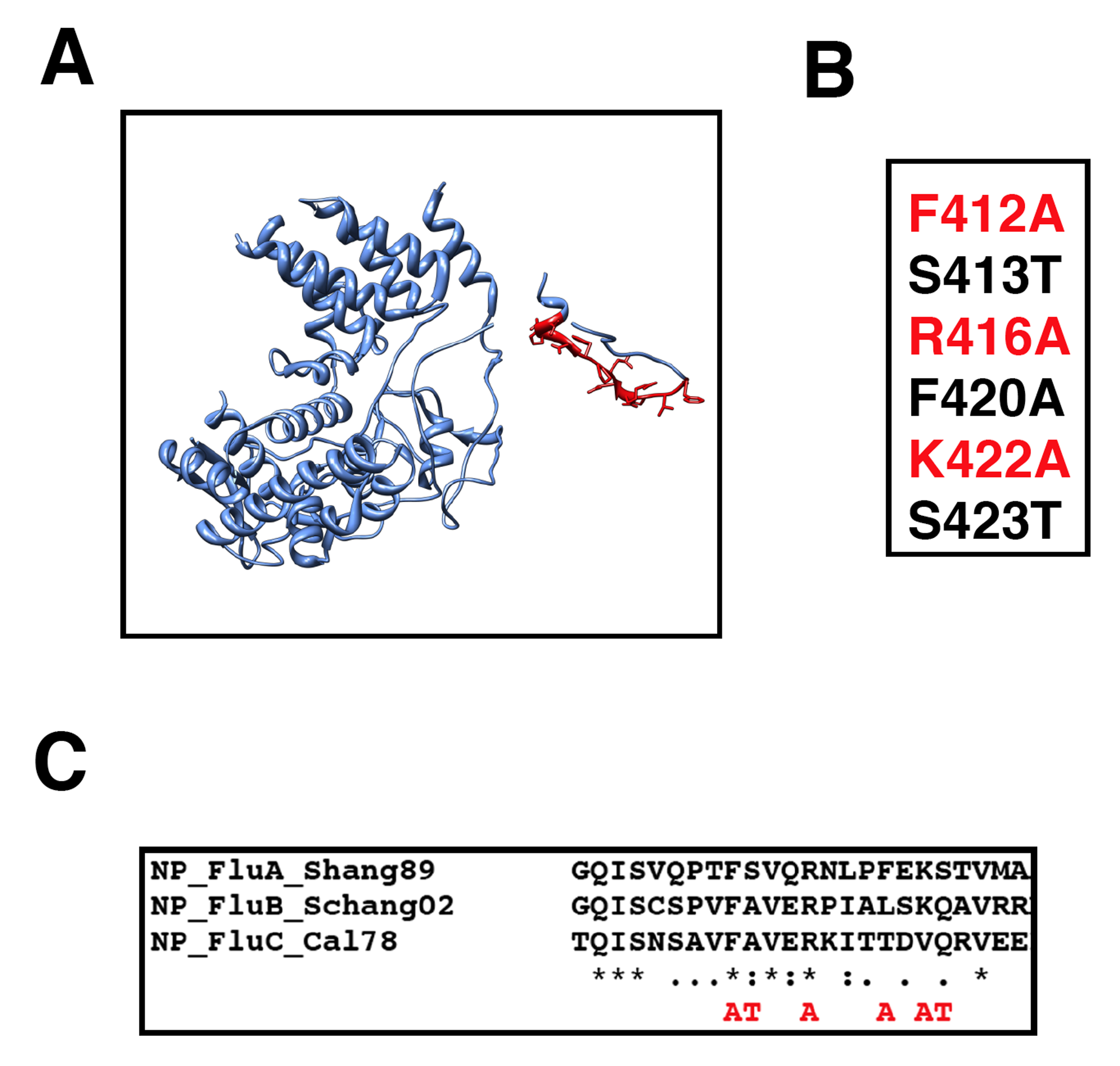

Supplement: Figure S5 — Mutations to analyse the NP-NP interaction site. (A) The atomic structure of influenza NP is shown in an orientation appropriate to see the protruding loop present around position 420 in the sequence. The relevant amino acids in the loop are highlighted. (B) The mutations introduced in the loop are indicated, including those involving non-conservative changes in conserved positions (in red) and conservative changes in non-conserved positions (in black). (C) Alignment of the relevant sequence for influenza viruses of the A, B and C types indicating the same mutations indicated in panel B. (1.60 MB TIF) [file ppat.1000491.s005.tif]
